# Prefrontal Function Engaging in External-Focused Attention in 5- to 6-Month-Old Infants: A Suggestion for Default Mode Network

**DOI:** 10.3389/fnhum.2016.00676

**Published:** 2017-01-10

**Authors:** Mingdi Xu, Eiichi Hoshino, Kiyomi Yatabe, Soichiro Matsuda, Hiroki Sato, Atsushi Maki, Mina Yoshimura, Yasuyo Minagawa

**Affiliations:** ^1^Department of Psychology, Faculty of Letters, Keio UniversityYokohama, Japan; ^2^Center for Life-Span Development of Communication Skills, Keio UniversityYokohama, Japan; ^3^Global Centre for Advanced Research on Logic and Sensibility, Keio UniversityTokyo, Japan; ^4^Graduate School of Human Relations, Keio UniversityTokyo, Japan; ^5^Center for Exploratory Research, Research and Development Group, Hitachi, Ltd.Hatoyama, Japan; ^6^Brain Science Business Unit, Innovation Promotion Division, Hitachi High-Technologies CorporationTokyo, Japan

**Keywords:** fNIRS, attention, prefrontal cortex (PFC), infant, default mode network (DMN)

## Abstract

The present study used functional near-infrared spectroscopy (fNIRS) to measure 5- to 6-month-old infants’ hemodynamic response in the prefrontal cortex (PFC) to visual stimuli differing in saliency and social value. Nineteen Japanese 5- to 6-month-old infants watched video clips of Peek-a-Boo (social signal) performed by an anime character (AC) or a human, and hand movements without social signal performed by an AC. The PFC activity of infants was measured by 22-channel fNIRS, while behaviors including looking time were recorded simultaneously. NIRS data showed that infants’ hemodynamic responses in the PFC generally decreased due to these stimuli, and the decrease was most prominent in the frontopolar (FP), covering medial PFC (MPFC), when infants were viewing Peek-a-Boo performed by an AC. Moreover, the decrease was more pronounced in the dorsolateral PFC (DLPFC) when infants were viewing Peek-a-Boo performed by an AC than by a human. Accordingly, behavioral data revealed significantly longer looking times when Peek-a-Boo was performed by an AC than by a human. No significant difference between Peek-a-Boo and non-Peek-a-Boo conditions was observed in either measure. These findings indicate that infants at this age may prefer stimuli with more salient features, which may be more effective in attracting their attentions. In conjunction with our previous findings on responses to self-name calling in infants of similar age, we hypothesize that the dynamic function of the MPFC and its vicinity (as part of default mode network (DMN): enhanced by self-focused stimuli, attenuated by externally focused stimuli), which is consistently observed in adults, may have already emerged in 5- to 6-month-old infants.

## Introduction

Humans are highly social creatures and develop communicative capacities via various interactions with others very early in life. Converging evidence from functional neuroimaging studies suggests that the medial prefrontal cortex (MPFC) plays a pivotal role in social cognition, especially self-related information processing, not only in adults, but also in young infants (Kampe et al., 2003; Amodio and Frith, 2006; Northoff et al., 2006; Andrews-Hanna et al., 2010; Grossmann, 2013; Imafuku et al., 2014). Interestingly, neural activity of dorsal MPFC (DMPFC) and its peripheral areas has been shown to increase in tasks involving self-referential attention, and to decrease in tasks involving externally focused attention (Gusnard and Raichle, 2001). This phenomenon has been suggested to be related to the default mode network (DMN), which serves as a baseline between self-referential and externally focused states (Gusnard and Raichle, 2001; Raichle et al., 2001; Buckner et al., 2008). Such dynamic functional patterns have been consistently found in studies on adult subjects. However, at which point during development of brain function these dynamic patterns first occur remains to be elucidated.

To date, research on DMN has mainly focused on adults. Although some recent developmental studies have reported novel findings on DMN in infants and children, a comprehensive understanding of DMN development, especially during the first year of life when the brain undergoes the most dramatic development (Gao et al., 2015), has not yet been attained. Fransson et al. (2007) did not detect a DMN in preterm infants at a gestational age of 41 weeks. Xiao et al. (2016) found developmental changes of DMN subsystems between 3- and 5-year-olds. de Bie et al. (2012) reported that DMN is present but less mature in awake 5- to 8-year-olds compared with older children and adults. Fair et al. (2008) found an only sparsely connected DMN in 7- to 9-year-olds. Gao et al. (2009) focused their research on children of younger age filling the gap between the above-mentioned studies: in a resting-state fMRI study on children between 2 weeks and 2 years of age, evidence for a primitive and incomplete DMN structure in 2-week-old infants and for an adult-like DMN in 1- and 2-year-old children was found. Recently, Gao et al. (2015) further investigated neural networks of infants from <1 month to 1-year-old in 3-month steps, and found that the structure of DMN undergoes major changes during the first 3 months of life, continues development during the first year of life and becomes adult-like at 1-year of age.

Taken together, these findings approximately sketch out the developmental trajectory of DMN structure from early life. However, the early development of DMN function remains unclear, because most developmental studies examined resting-state (sleeping) children to effectively collect fMRI data. Based on findings of Gao et al. (2015) that the long distance synchronization of two typical hubs of the DMN, i.e., the MPFC and the posterior cingulate cortex (PCC), showed significant strengthening during the second half of the first year, we focused our infant functional studies on this critical age for structural development. To elucidate whether the aforementioned dynamic function of PFC has already emerged at this early age and to shed light on the functional development of primitive DMN, we designed two distinct sets of experiments. The first experiment, published in a previous report, focused on self-referential tasks and found increased hemodynamic responses in the DMPFC in 6-month-old infants in response to self-name calling (Imafuku et al., 2014).

In the present study, we focused on externally focused attention tasks in 5- to 6-month-old infants. Infant-directed social communication cues—Peek-a-Boo video clips performed either by a Japanese woman or an anime character (AC) “ANPANMAN”, and video clips without Peek-a-Boo performed by an AC—were presented to healthy Japanese 5- to 6-month-old infants. Functional near-infrared spectroscopy (fNIRS) was used to measure the activity of PFC in infants while their behavior (looking time) was recorded simultaneously. If the functional activation in response to externally-directed (non-self-referential) stimuli of the PFC of 5- to 6-month-old infants resembled that of adults, we would expect an attenuated cerebral response in PFC elicited by stimuli with Peek-a-Boo. In addition, we predict that PFC activity may depend on other properties (saliency and social value) of the stimulus. Specifically, Peek-a-Boo performed by an AC might induce a more prominent hemodynamic response than Peek-a-Boo performed by a human, because of the saliently emphasized facial features of the AC (exaggerated facial parts, high brightness and strong contrast); and than non-meaningful hand-waving movements performed by an AC because of the social value of Peek-a-Boo.

## Materials and Methods

### Participants

Nineteen 5- to 6-month-old Japanese infants (9 males and 10 females; mean age: 173.2 ± 14.3 (150~196) days old) participated in this study. Another nine infants participated in the study, but were excluded from final analysis because of insufficient valid trials (at least three valid trials for each condition) attributable to extensive fussiness, motion artifacts or technical problems. All participants were full term infants at birth and had no history of serious diseases or disorders. All parents volunteered by responding to advertisements and were paid for participation of their child. Both parents of all infants’ parents were Japanese. Informed consent was obtained from the parents before the study. The study protocol was approved by the Ethics Committee of Keio University, Faculty of Letters (No. 120223-1) and was in accordance with the latest version of the Declaration of Helsinki.

### Stimuli

**Figure 1:**
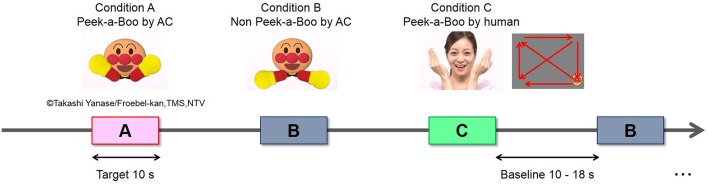
**Experimental design and stimuli**.

Full-color, life-size video clips of Peek-a-Boo performed by a human woman or an AC (“ANPANMAN”) were used as social stimuli. The AC used in this study has exaggerated facial and body parts (eyebrows, eyes, cheeks, nose, mouth and hands), strong contrast, and high brightness (Figure 1). Other features of the AC and the human woman (size and movements of face and hands) were controlled to be comparable as much as possible. Vocal signals expressing Peek-a-Boo were recordings of a female voice actor speaking Japanese and were identical for AC and human Peek-a-Boo conditions. During the experiment, the vocal sound amplitude was adjusted to be around 70 dB SPL measured at the approximate location of each infant’s head. Three types of video clips were used as experimental conditions: (A) Peek-a-Boo performed by an AC; (B) non-meaningful hand-waving movements performed by an AC; and (C) Peek-a-Boo performed by a human woman. A small-sized AC face moving slowly on a blank background served as the baseline. This stimulus contains no social signal and is able to maintain the infants’ gazes at the screen.

### Experimental Procedure

Prior to the experiment, all infants were screened for typical cognitive functioning using the Kyoto Scale of Psychological Development (KSPD; Ikuzawa et al., 2002). In addition, the infants’ familiarity with the AC and Peek-a-Boo was examined. All questionnaires were completed by the infants’ parents.

During the experiment, infants were seated on the lap of their mothers in a sound-attenuated chamber. Mothers wore headphones to prevent them from hearing the stimuli during the experiment. Video stimuli were presented at a viewing distance of approximately 40 cm on an 18.1″ LCD monitor controlled by a computer outside the sound-attenuated chamber. The loudspeaker was mounted on the monitor at about the height of the infant’s head. Infants were encouraged to focus on the displayed stimuli, and experiments were terminated when infants became fussy or bored. A video camera was set behind the monitor to record the infants’ behavior (e.g., body and eye movements) during the experiment. An experimenter outside the sound-attenuated chamber observed the infant’s behavior during the experiment via another monitor connected to the video camera to monitor the progress of the experiment.

The experiment was block-designed. The sequence of stimulus presentation is shown in Figure 1. One experimental trial made up of a baseline period of 10~18 s (randomly) and a target period of 10 s. Each of the three conditions was presented in seven target blocks. The total of 21 trials was pseudo-randomly arranged with the constraint that no two consecutive trials were of the same condition. Moreover, the presentation order of the 21 trials was counterbalanced across participants. The total time of the whole experiment was around 7~9 min. Presentation of video stimuli was controlled using Visual Basic 6.0.

### Data Acquisition

The infants’ behavior was recorded on a DVD throughout the experiment for later assessment of movement artifacts and looking time at the stimulus. Hemodynamic responses in the PFC region were recorded using a multichannel NIRS system (ETG-7000, Hitachi Medical Co., Japan). The system emits continuous near-infrared lasers with fixed wavelengths of approximately 780 nm and 830 nm. Lasers are modulated at different frequencies depending on the wavelengths and the channels, and are detected using lock-in amplifiers (Watanabe et al., 1996). The device provides estimates of changes in hemoglobin (Hb) concentrations and their oxygenation levels of the optical paths in the underlying brain region between the nearest pairs of emitter and detector probes.

A silicon probe pad was used to arrange eight emitters and seven detector probes in a 3 × 5 rectangular lattice, forming 22 recording channels. Each pair of emitter and detector probes was separated by 20 mm, and the spatial resolution was estimated to be 15~30 mm. It has been suggested that near-infrared light penetrates deeper into the cortex in infants than adults, because infant brains contain less myelinated and less reflective white matter (Fukui et al., 2003). Therefore, the 20 mm distance between each emitter and detector probes pair used in this study was able to capture cerebral activity in relatively deeper regions of infant PFC, around 20~35 mm from the scalp-skin surface (Imafuku et al., 2014).

**Figure 2:**
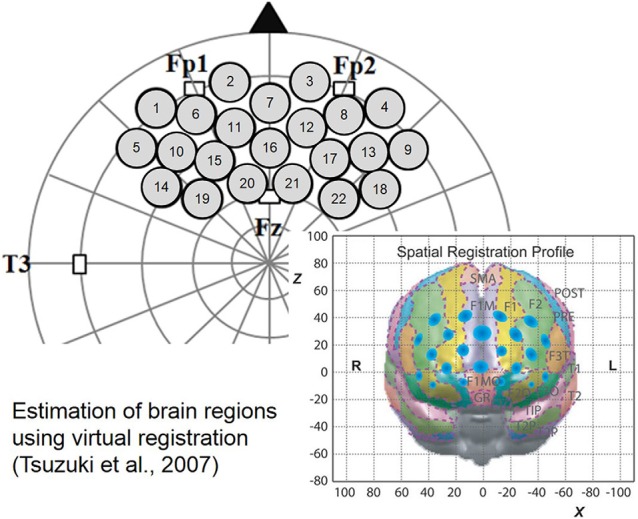
**Probe set and location on the brain**.

The 3 × 5 probe pad was placed on the infants’ PFC region as shown in Figure 2. Specifically, the bottom border of the probes was placed in a direction horizontal to the line connecting T3, Fp1, Fp2 and T4 on the international 10-20 system, and the center of the channels was positioned across the nasion–inion line (Jasper, 1958). This probe arrangement enabled us to estimate the specific brain region of any localized cerebral activity based on the virtual registration method (Tsuzuki et al., 2007). After probe placement, the experimenter verified that each probe was in adequate contact with the scalp. Only after this verification NIRS recordings were initiated.

### Behavioral Data Analysis

**Figure 3:**
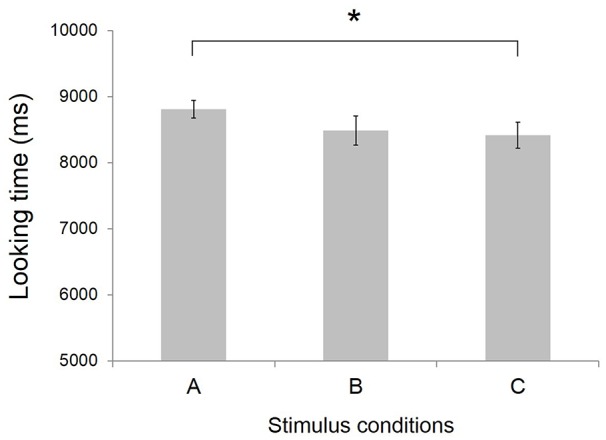
**Mean looking time at video clips stimuli (*N =* 17).** Error bars indicate standard error of the mean. *p < 0.05. Conditions definition: **(A)** Peek-a-Boo by anime character (AC); **(B)** Non Peek-a-Boo by AC; and **(C)** Peek-a-Boo by human.

The recorded DVDs were used for the looking time analysis. Infants’ gaze durations toward video clip stimuli during both baseline and target periods were estimated by two trained coders at 100 ms intervals using the behavior coding software GenobsX. Data from two infants was excluded from further analysis because of technical problems. Valid behavioral data was obtained from 17 infants (Figure 3).

### NIRS Data Analysis

NIRS data were analyzed using Platform for Optical Topography Analysis Tools (POTATo) developed by Research and Development Group, Hitachi, Ltd. in a Matlab 7.7 environment (The MathWorks, Inc., Natick, MA, USA). Changes in concentration of oxygenated (oxy-) Hb and deoxygenated (deoxy-) Hb were calculated from absorbance changes of 780 nm and 830 nm laser beams sampled at 10 Hz. For each participant, the raw oxy- and deoxy-Hb data in each channel were bandpass filtered (Butterworth) at 0.02–0.7 Hz separately for each trial to remove components of physiological nature (e.g., respiratory and cardiac activity) and body movements (Taga et al., 2003; Naoi et al., 2012). Trial rejection was based on two criteria. First, based on analysis of the behavioral data, any block containing failure of gazing, speaking or body movements exceeding 5 s in total was discarded. Second, any block containing oxy-Hb changes larger than 0.15 mM/mm within 200 ms was discarded. In addition, we also discarded the data of the nine lower channels (CH1–CH9) because the amplitudes of the changes in Hb concentrations were too large, synchronous and in the same direction, indicating loose attachment of probes in the lowest line. The loose attachment might have been caused by unfitted silicon probe pad against the lower part of the infant’s forehead.

**Figure 4:**
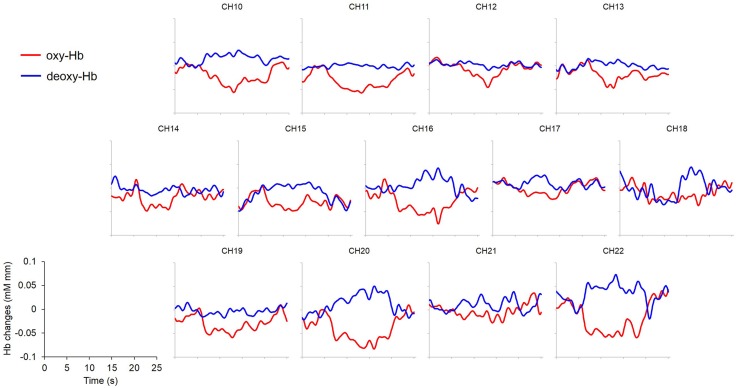
**Grand averaged (*N = 19*) time courses of changes in oxy- and deoxy-Hb in response to condition (A) Peek-a-Boo by AC as an example.** Channels 1~9 were not displayed because of excessive noises due to poor attachment to the infants’ foreheads. Red lines: change of oxy-Hb; blue lines: change of deoxy-Hb. *x*- and *y-axis* scales and labels are shown in the lower left corner. Stimuli requiring externally focused attention elicited widespread decrease in oxy-Hb.

Further analysis focused on a 20 s epoch composed of 5 s pre-target baseline, 10 s target block and 5 s post-target. For each condition, Hb concentrations of all valid trials were averaged. Subsequently, a time course of the mean change in oxy-Hb and deoxy-Hb was compiled for each channel, condition and participant. These average time courses for each participant were then grand averaged to form time-dependent waveforms of the hemodynamic responses in each channel under each condition (Figure 4).

Peak Hb changes were observed approximately 5~10 s after target stimulus onset across infants and channels. Therefore, we defined this period of interest as analysis window. For statistical analysis, mean oxy-Hb concentration changes during this time window were calculated and compared with mean oxy-Hb concentration changes during the 5 s pre-target baseline period for each channel, condition, and participant. To determine activated brain areas in each condition, differences in the changes of oxy-Hb concentrations between target and baseline periods were compared using a two-tailed paired *t*-test for each channel. False discovery rate (FDR) was used to correct for multiple comparisons across all channels. Brain regions underlying each channel were estimated using the virtual registration for NIRS channels (Okamoto et al., 2004; Okamoto and Dan, 2005; Tsuzuki et al., 2007). This method has been used consistently in the fNIRS literature (e.g., Minagawa-Kawai et al., 2009; Dan et al., 2013; Imafuku et al., 2014). A detailed account of the reliability of this method, especially for infants, can be found in our previous study (Imafuku et al., 2014). Further, to detect any difference in response between conditions, we applied a one-way analysis of variance (ANOVA) to the mean oxy-Hb concentration changes in the target period for each channel, with condition (Peek-a-Boo by AC vs. No Peek-a-Boo hand movements by AC vs. Peek-a-Boo by human) as within-subject factor. To control for between-condition discrepancies in global response magnitude, zero correction was performed on the response in the target period before ANOVA. Specifically, the response at the time of stimulus onset was adjusted to be zero for each condition. The tukey *post hoc* test was used to further clarify any significant main effects.

Last, correlations between the infants’ behavioral responses and hemodynamic responses measured with NIRS were assessed. Pearson’s correlation coefficients were calculated between the behavioral results (looking time, developmental score, familiarity with the AC and Peek-a-Boo) and the oxy-Hb concentration changes in significant channels.

## Results

### Behavioral Results

As shown in Figure 3, the mean looking time was 8.81 ± 0.13 s (mean ± SEM) for condition A Peek-a-Boo by AC, 8.49 ± 0.22 s for condition B No Peek-a-Boo hand movements by AC, and 8.42 ± 0.20 s for condition C Peek-a-Boo by human. A one-way ANOVA with stimulus condition as within subject factor showed a tendency for a main effect of stimulus condition (*F*_(2,16)_ = 2.58, *p* = 0.092). *Post hoc* tests revealed that infants’ looking times at condition A was significantly (*t* = 2.07, *p* < 0.05) longer than at condition C, indicating the infants’ preference for Peek-a-Boo performed by an AC over Peek-a-Boo performed by a human.

### NIRS Results

An overall decrease in oxy-Hb concentration was observed in PFC areas regardless of condition. However, the decrease was of larger magnitude and was more widely spread in condition A Peek-a-Boo made by AC than in the other conditions (Figure 4).

**Figure 5:**
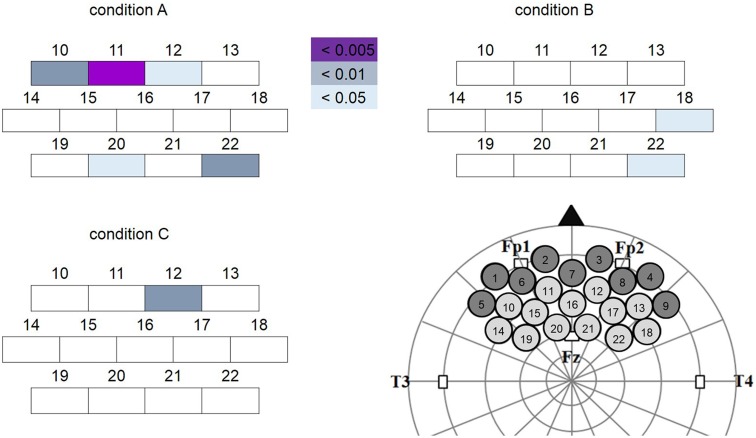
**Channels showing significant oxy-Hb decrease (target vs. baseline) in each condition.** The colored boxes represent channels showing significant oxy-Hb decrease at different levels (purple: significant channel after false discovery rate (FDR) correction). Channels 1~9 were excluded from analysis because of excessive noise. Location of each channel on the brain was illustrated in the lower right corner. Note that the oxy-Hb decrease was more widespread in condition A (Peek-a-Boo by AC) than in conditions B (Non Peek-a-Boo by AC) and C (Peek-a-Boo by human).

Two-tailed paired *t*-tests were performed on the data as described in the “*NIRS Data Analysis*” Section. This analysis revealed different but overlapping channels showing a significant decrease in oxy-Hb concentration change in response to the different conditions. Specifically, oxy-Hb concentration change was significantly decreased in channels CH10, CH11, CH12, CH20 and CH22 for condition A; CH18 and CH22 for condition B; and CH12 for condition C (Figure 5). Using virtual registration for NIRS measurement, the corresponding brain regions for these channels were estimated to be frontopolar (FP), dorsal lateral PFC (DLPFC) and DMPFC for condition A; right DLPFC for condition B; and FP for condition C, respectively. After FDR correction for multiple comparisons, only oxy-Hb decrease in CH11 in condition A was significant (colored in purple in Figure 5). The corresponding estimated brain region of this channel was FP (FP 53%, DLPFC 47%). Please note that in infants, NIRS measurement captures hemodynamic responses from deeper regions in addition to the superficial part of cerebral cortex, owing to less myelinated and less reflective white matter (Fukui et al., 2003). Therefore, in case of the infants in our study, responses of deeper regions like the MPFC are likely to have formed part of the significant decrease of oxy-Hb response allocated to FP.

**Figure 6:**
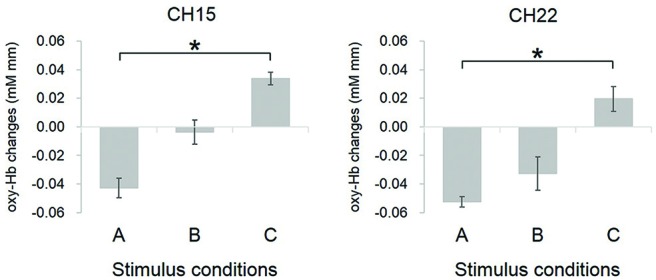
**Comparison of oxy-Hb changes in channels showing significant difference between conditions.** Conditions definition: **(A)** Peek-a-Boo by AC; **(B)** Non Peek-a-Boo by AC; and **(C)** Peek-a-Boo by human. For both CH15 and CH22, the oxy-Hb response in condition A was significantly more decreased as compared to condition C. Error bars indicate one standard error of the mean (*N* = 19). *p < 0.05.

Furthermore, one-way ANOVA (with condition as within subject factor) performed on each channel revealed that CH15 (*F*_(2,54)_ = 4.73, *p* = 0.01) and CH22 (*F*_(2,54)_ = 3.59, *p* = 0.04) showed significant differences of oxy-Hb decrease between conditions (Figure 6). The estimated brain regions of these two channels were DLPFC for CH15 (DLPFC 65%, DMPFC 30%, FP 5%) and DLPFC for CH 22 (DLPFC 97%, DMPFC 3%). Tukey *post hoc* tests confirmed that both for CH15 (*p* = 0.01) and CH22 (*p* = 0.03), oxy-Hb decrease was significantly larger in condition A than in condition C, indicating that infants focused their attention more easily on Peek-a-Boo performed by an AC than by a human, and that DLPFC may reflect the degree of externally focused attention.

Finally, no significant correlation was found between oxy-Hb changes and behavior (looking time, developmental score, or familiarity with AC and Peek-a-Boo) in any channel.

## Discussion

The present study employed behavioral and fNIRS recordings to examine 5- to 6-month-old infants’ cerebral responses to externally focused stimuli with different social value (with or without Peek-a-Boo) and stimulus saliency (human or AC). Behavioral data showed that infants spent significantly more time looking at Peek-a-Boo performed by an AC than at Peek-a-Boo performed by a human, indicating more interest in the AC. The increased interest for the AC relative to the human may have been caused by the more salient facial features of the AC. NIRS data revealed that the hemodynamic response in the infant PFC generally decreased for the externally focused stimuli employed in this study. Peek-a-Boo performed by an AC induced the most widespread decrease among the three conditions and the decrease was most significant in FP including MPFC. Moreover, a significantly larger decreased response was observed when the infants viewed Peek-a-Boo performed by an AC than by a human in the DLPFC. We will discuss the implications of these decreased hemodynamic responses later on in this article.

Since the development of neuroimaging methods, especially resting state fMRI (rsfMRI), an increasing number of studies focused on the structural development of the infant brain. However, functional studies of infants under 1-year of age (the most vital period for brain development) are scarce owing to the difficulty to make such young infants attend to certain stimuli obediently and motionlessly. Motion is detrimental to most neuroimaging methods. fNIRS is a promising method to investigate brain functions of children and infants because it is much lesser vulnerable to body and head motion. In addition, fNIRS is exceptionally safe. Functional studies of the infant brain using fNIRS have accumulated in recent years. However, studies investigating higher-order functions such as social cognition are still rare. A recent fNIRS study demonstrated increased response to social stimuli when compared to non-social stimuli in the temporal area (superior temporal sulcus (STS)) in 5-month-old infants, resembling the functional specialization in adults (Lloyd-Fox et al., 2009). Similar to the current study, this previous study used Peek-a-Boo performed by a human as a social stimulus, suggesting suitability of Peek-a-Boo to attract attention and serve as a social communicative cue for infants as young as 5-month old.

Our study employed similar social stimuli, but we focused on the cerebral responses of infants in another key region involved in social cognition, the MPFC (Grossmann, 2013). Moreover, we aimed not only to pinpoint the specialized region in the PFC responsible for processing social stimuli, but also to investigate how 5- to 6-month-old infants respond to stimuli that require externally focused (non-self-referential) attention, and whether responses recorded from infants resemble responses recorded from adults. Our fNIRS measurements demonstrated reduced PFC activity in response to stimuli that required externally focused attention, similar to previous neuroimaging studies on adults (Shulman et al., 1997; Gusnard and Raichle, 2001). Gusnard and Raichle (2001) have discussed such task-induced but task-independent decreases in PFC activity in depth based on the findings of several PET studies in adults. These functional studies employed stimuli from different domains involved in a wide variety of tasks, such as motoric activity, language processing, visual and auditory attention, etc. (Shulman et al., 1997; Mazoyer et al., 2001). However, the decreased responses hardly varied in their locations, suggesting the existence of a systematized mode of brain function—the default mode (Raichle et al., 2001), which is attenuated during externally directed behaviors. Furthermore, as Gusnard and Raichle (2001) have concluded from a number of functional neuroimaging studies, the DMPFC, a key part of the DMN (Gao et al., 2009; Andrews-Hanna et al., 2010; Xiao et al., 2016), may encompass a dynamic range of activity that is enhanced in tasks that involve self-referential mental activity (Castelli et al., 2000; Gusnard et al., 2001), and attenuated in tasks that require externally directed (non-self-referential) attention (Shulman et al., 1997).

Our group confirmed the presence of such dynamic functional activity in DMPFC and its vicinity of infants as young as 5- to 6-month of age. Specifically, our previous fNIRS study (Imafuku et al., 2014) demonstrated increased activity in the DMPFC of 6-month-olds (185 ± 21.9 days) in response to self-referential stimuli (self-name calling). Using the same instrument and probe arrangement, the present fNIRS study revealed that externally directed stimuli decreased responses relative to baseline in the PFC region including FP, DMPFC and DLPFC of infants of similar age (173.2 ± 14.3 days). This data indicates primitive DMN functioning in 5- to 6-month-old infants. rsfMRI studies in young infants lend further support to this possibility: Gao et al. (2009) found primitive DMN structure in 2-week-old infants, and approximately adult-like DMN structure, in particular the two key hubs (MPFC and PCC), in 1-year-old infants. Furthermore, Gao et al. (2015) suggested that DMN structure undergoes the most prominent development during the first 3 months of life and that the long-distance connectivity of MPFC and PCC strengthens to a large extent during the second half of the first year.

One may wonder: (1) why the supposed infant DMN structure reported here is so large, including not only MPFC, but also lateral frontal regions like DLPFC, which is known as a central region of executive control (MacDonald et al., 2000); and (2) how our study aligns with previous studies that have shown increased rather than decreased hemodynamic responses induced by attention-directing cues in DLPFC (e.g., Hopfinger et al., 2000).

Regarding the dynamic pattern of activity in infant DMN structure, two possible reasons may explain the seemingly broader activated region. First, even in studies of adult subjects, the decreased activity in response to externally focused attention covered a larger region including not only the DMPFC but also adjacent areas like the ventral MPFC (see Figure 5 from Raichle et al., 2001; Figure 4 from Gusnard et al., 2001; and Figure 1 from Gusnard and Raichle, 2001). In our study, after FDR correction for multiple comparisons, only response in CH11 in condition A was significantly decreased when compared to baseline. The estimated region for CH11 is FP, which includes partial ventral MPFC. Second, both in our present and previous infant studies using NIRS, the regions underlying each significant channel were estimated based on the virtual registration method for NIRS channels (Okamoto et al., 2004; Okamoto and Dan, 2005; Tsuzuki et al., 2007). This method principally projects superficial parts of cerebral cortex. NIRS light penetrates much deeper into the brains of infants than into the brains of adults owing to infants’ less myelinated and less reflective white matter in addition to much thinner skull and scalp (Fukui et al., 2003). Therefore, the estimated DLPFC activity in the present study may also contain activity from deeper regions including DMPFC. According to these interpretations, the dynamic pattern of DMN activity is not necessarily restricted to DMPFC, at least for the decrease in activity accompanying externally focused attention. Therefore, we suggest that the dynamic DMN function observed in 5- to 6-month-old infants in our present (externally focused attention) and our previous (self-focused attention) study (Imafuku et al., 2014) is comparable to the dynamic DMN function observed in adults, which has been concluded from several independent functional neuroimaging studies as well (Shulman et al., 1997; Gusnard and Raichle, 2001; Gusnard et al., 2001; Raichle et al., 2001).

Regarding DLPFC activity, the decrease in the hemodynamic response was significantly larger for more salient Peek-a-Boo stimuli (larger for the AC than the human), indicating that DLPFC modulation may reflect the degree of attention. Many studies have found increased DLPFC hemodynamic response in attention tasks (e.g., Hopfinger et al., 2000). However, brain regions may respond differently to different task demands. In the present study, we may have found decreased hemodynamic responses because we used a passive viewing task for 5- to 6-month-old infants who are not able to perform attentional tasks at such a young age. Tasks requiring explicit execution may be quite another matter.

Interestingly, decreased hemodynamic responses were found to be more prominent when Peek-a-Boo was performed by an AC rather than by a human woman. Compared to the human face, the facial parts (eyebrows, eyes, cheeks, nose and mouth) of the AC were exaggerated; figure and color were emphasized and the AC showed a higher level of brightness and contrast. These features made the AC face a more salient stimulus than the human face. Previous research has reported that the vision of young infants is not fully developed until 3-years of age (Held, 1979; Norcia and Tyler, 1985; Maurer and Lewis, 2001). Therefore, it is possible that stimuli with more outstanding features are best suited to attract infants’ attention. In fact, face stimuli with salient features were found to be more attractive to infants at early developmental stage and such stimuli elicited strong activation in their frontal regions (Perkins, 1975; Rhodes et al., 1987; Ellis et al., 1992). The behavioral results of our study support this possibility: the infants spent significantly more time looking at Peek-a-Boo performed by an AC than at Peek-a-Boo performed by a human. Based on these reasons, we suggest that the DLPFC, where the most prominent difference in activity was found between conditions (A vs. C), may reflect the degree of attention focused on external stimuli by infants as young as 5–6 months old. Moreover, we propose that everyday learning may be facilitated in infants of this young age by using stimuli with salient features.

The present experimental design used Peek-a-Boo performed by an unknown woman. We hypothesize that if Peek-a-Boo was performed by the infants’ mothers, the decrease in response in the infant’s PFC would be strongest to the mother’s Peek-a-Boo. Previous behavior and neuroimaging literature suggests that the mother is extremely important to infants’ early life (Minagawa-Kawai et al., 2009; Imafuku et al., 2014). Therefore, mother-related stimuli may be special to young infants and elicit unique brain activity. We are planning to test this hypothesis in our future investigations.

In addition, to keep the infants’ attention on the display, we presented a small-sized moving AC face rather than a blank screen with a cross mark (as used in studies on adult subjects) for fixation during the baseline period. One might suspect that the eye movements induced by this moving AC face might have activated the DPFC (e.g., frontal eye field) and have led to the decreased activity during the target period relative to the baseline period. Indeed, we tried to exclude such interference as far as possible: (1) movement of the small-sized AC face was much slower than the Peek-a-Boo movement during the target period; and (2) the range of movement of the small-sized AC face was controlled to be identical to the range of the Peek-a-Boo movement in the target period. Based on these precautions, we assume that eye-movement was comparable between baseline and target periods. Moreover, behavioral data showed that the mean looking time of infants was 6.43 ± 0.34 s (mean ± SEM) during the 10~18 s baseline period, much shorter than that during the 10 s target period under the three different conditions (8~9 s). Therefore, we conclude that the decreased hemodynamic responses in the PFC reflect externally focused attention rather than eye movements during the baseline period.

Last, we did not observe a significant difference between social and non-social stimuli performed by an AC, neither in the behavioral nor in the NIRS measurement. In contrast, Lloyd-Fox et al. (2009) found significantly more activation in response to social stimuli than to non-social stimuli in STS of infants of similar age. However, our study focused on a different brain region (the PFC). Therefore, our results do not need to be consistent with these previous findings. Rather, this discrepancy may reflect that 5- to 6-month-old infants have developed specialized regions for processing of social stimuli including the STS but not the PFC.

## Conclusion

In this study we used fNIRS, a suitable and promising instrument for infant functional study, to investigate hemodynamic responses to tasks that require externally focused attention in 5- to 6-month-old infants. Our NIRS data revealed decreased PFC responses elicited by externally directed stimuli depending on stimulus property. Stimuli with more salient features induced decrease in response to a larger extent. In conjunction with our previous findings on enhanced PFC responses in infants of similar age performing tasks using self-referential stimuli (self-name calling), we suggest primitive functioning of DMN in 5- to 6-month-old infants.

## Author Contributions

HS, AM, MY, SM and YM designed research; YM performed research; EH, KY and YM analyzed data; and MX and YM wrote the article.

## Conflict of Interest Statement

The authors declare that the research was conducted in the absence of any commercial or financial relationships that could be construed as a potential conflict of interest.
